# Social capital and community disaster resilience: post-earthquake tourism recovery on Gili Trawangan, Indonesia

**DOI:** 10.1007/s11625-020-00854-2

**Published:** 2020-09-03

**Authors:** Stefan Partelow

**Affiliations:** Leibniz Centre for Tropical Marine Research (ZMT), Fahrenheitstrasse 6, 28359 Bremen, Germany

**Keywords:** Natural disaster, Natural hazard, Earthquake, Collective action, Tourism, Tropical, Coastal

## Abstract

This study examines the hypothesis that social capital can be a foundation for community disaster resilience with an analysis of empirical findings from the August 2018 earthquake recovery on Gili Trawangan, Indonesia, a globally known coastal tourism destination. Positive links between community social capital and community disaster resilience are hypothesized, but the extent to which an iterative and interdependent relationship is co-shaping both is less understood. Social capital can enable collective action, providing self-organized social, psychological, financial and material resources following a disaster, that may otherwise need to be provided externally. In turn, disasters create collective action problems where collective response and recovery process are needed, creating an institutional space where the degree of usefulness, meaning and function of social capital can be shaped, recognized and drawn upon, often where external aid is insufficient. These relationships can be observed following disasters, because individuals and communities are often linked through emotive and meaningful sequences of common experiences, actions and activities. Findings descriptively detail Gili Trawangan’s response and recovery process through the events and activities that occurred, and are then theoretically analyzed with a social capital framework including bonding (within group), bridging (between group) and linking (across power and institutional) ties. Three conclusions can be summarized. (1) Community social capital and disaster resilience are iteratively co-shaped through collective experiences, actions and activities. (2) Understanding context is critical for understanding if and to what degree this relationship exists. (3) The mechanism through which social capital enhances resilience is that it can enable collective action that can lead to the provision of needed aid and services.

**Electronic supplementary material:**

The online version of this article (10.1007/s11625-020-00854-2) contains supplementary material, which is available to authorized users.

## Introduction

October 2018—Gili Trawangan, Indonesia. Stepping off the boat, the often chaotic but vibrant tourism island appeared, surprisingly, as it had during previous visits in April 2017 and October 2015. Less than 90 days earlier, the island endured four severe earthquakes^1^ (6.4 (July 28, 2018), 6.9 (August 5, 2018), 6.5 (August 19, 2018), 6.9 (August 19, 2018), with over 700 aftershocks in the following 3 months (USGS 2018). However, if you were a first-time tourist, the island may appear as advertised. Many SCUBA businesses and accommodations were open and the island’s reputation as a top destination for vacation and diving was on offer. Despite nearly 4 weeks of evacuations and closure throughout August 2018, the island’s initial recovery seemed rapid.

Disaster studies (Lindell 2013) has increasingly focused on the role of social organization, including social capital in preparedness, response and recovery processes (Dynes 2005; Aldrich 2010; Lindell 2013). Many events can lead to disasters including terrorism, political oppression, disease epidemics, arson, earthquakes, flooding, wildfires or hurricanes. The spatial impact of disasters can characterize an event as well as event frequency (e.g., earthquakes plus aftershocks or a single tsunami). Characterizing recovery processes can be both short-term (e.g., immediate medical or humanitarian needs; clearing debris) and long-term impacts (e.g., environmental contamination; psychological trauma; community and economic rebuilding). A critical point is that “disasters occur when the negative effects of the hazards are not well managed (p. 101),” (Sanyal and Routray 2016), emphasizing that disaster risk reduction is a function of the factors that enable preparedness, response and recovery.

Disaster preparedness, and what is needed for long-term post-disaster recovery, is almost exclusively framed by governments as a need for material capital, financial aid and physical infrastructure needs (Aldrich and Meyer 2015; Sadri et al. 2017), as framed in the United States (National Research Council 2012) and internationally in the Sendai Framework for Disaster Risk Reduction (United Nations 2015). Although the politics of infrastructures is a niche of its own e.g., (Barker 2017), what is less emphasized in mainstream political discourse are the social infrastructures, the social capital of communities representing the value of their collective civic, cultural and political realities. Such social capital, as detailed below, can play a substantial role in enabling community-based disaster response and recovery processes (Nakagawa and Shaw 2004; Sadeka et al. 2015; Sadri et al. 2017; Masud-All-Kamal and Monirul Hassan 2018; Wei and Han 2018; Gallagher et al. 2019). It is often social capital (e.g., often examined through network analysis) that enables access to the material infrastructures (Aldrich and Meyer 2015), and it can be social capital that creates a foundation for community resilience to disasters as a form of ‘social preparedness’. Aldrich and Meyer (2015) further explain that immediately following disasters, “social capital networks provide access to various resources…including information, aid, financial resources, and child care along with emotional and psychological support (p. 256),” and argue that many governments have focused only on physical infrastructure, “…despite evidence that social, not physical, infrastructure drives resilience (p. 254).” Furthermore, it is important to reflect on how the degree of community self-dependency influences social capital formation and its usefulness, both at the individual and community level.

Defining resilience is critical for analyzing disaster response and recovery, and understanding the analysis in this study. Norris et al. (2008) define community resilience as “a process linking a set of networked adaptive capacities to a positive trajectory of functioning and adaptation in constituent populations after a disturbance”. Complimentarily, Aldrich and Meyer (2015) define community resilience as “…the collective ability of a neighborhood or geographically defined area to deal with stressors and efficiently resume the rhythms of daily life through cooperation following shocks”. Considering these, disaster resilience in this study is defined as the ability “to resume the rhythms of daily life through cooperation”, (Aldrich and Meyer 2015).

On post-earthquake Gili Trawangan, resuming the rhythms of daily life is premised on the return of coastal tourism. However, coastal areas are vulnerable to many events that could lead to disasters, including anomalous weather and sea level rise (Adger et al. 2005; Adger 2010; Benevolenza and DeRigne 2019). Many tropical coastal tourism areas are rural and lack protective or preventative infrastructure (e.g., well-constructed buildings, emergency protocols, storm barriers, diverse food and water supply chains), making it more difficult to manage negative impacts. The “mobilization of assets, networks, and social capital [are needed] both to anticipate and to react to potential disasters [in vulnerable coastal areas]”, (p. 1037) (Adger et al. 2005). Social capital may be one of the few resources available for rural communities who have limited preventative infrastructure or access to external resources and aid (Sanyal and Routray 2016) (see (Cattell 2001) in the context of health).

### Social capital and disaster resilience

Concepts of social capital have been explored since the late 1800s (Lin 1999) and early 1900s (Hanifan 1916), and more explicitly as human or social capital in social organization theories such as collective action, social wellbeing and economic development since the 1960s (Woolcock 1998). Woolcock and Narayan (2000) define social capital as “…the norms and networks that enable people to act collectively (p. 226)”. An extended definition states that “…a person's family, friends, and associates constitute an important asset, one that can be called on in a crisis, enjoyed for its own sake, and leveraged for material gain. What is true for individuals, moreover, also holds for groups (p. 226)”.

Scholarship on social capital has examined multiple levels on the scale of social organization (e.g., individual, local community, national) (Lin 1999). Putnam (1995) notoriously argues that declining social capital in the United States is “…the single most important problem in America”, and that “…life is easier in a community blessed with a substantial stock of social capital….[and that] networks of civic engagement foster sturdy norms of generalized reciprocity and encourage the emergence of social trust….coordination and communication, amplify reputations, and thus allow dilemmas of collective action to be resolved (p. 66)”. At the community level, social capital has been studied extensively in environmental governance (Rudd 2000; Ostrom and Ahn 2001; Pretty and Ward 2001), social movements (Benford and Snow 2000; Lubell 2002), development (Woolcock and Narayan 2000), and disaster studies (Marín et al. 2015; Sadri et al. 2017; Wei and Han 2018; Gallagher et al. 2019; Li and Tan 2019). Pretty and Ward (2001) argue that social capital “….is central to equitable and sustainable solutions to local development problems…[and]…is likely to be related to the availability of social capital locally, but also to appropriate inputs from government and voluntary agencies”.

Nonetheless, social capital is a multidimensional concept, and numerous frameworks have helped define its constituent components to be measured. Szreter and Woolcock (2004) in support from other authors e.g., (Kawachi et al. 2004; Almedom 2005; Newman and Dale 2007; Aldrich and Meyer 2015) suggest “a more comprehensive but grounded theory of social capital,” developing a framework that includes (a) bonding, (b) bridging and (c) linking social capital (Table 1). Almedon (2005) adds to this framework, highlighting nested sub-concepts, including both structural (social networks) or cognitive dimensions (social control/efficacy; shared values; mutual trust and norms of reciprocity). Distinguishing types of social capital is useful for guiding empirical research and examining aggregate social mechanisms driving its formation. Frameworks allow for comparison between cases where context is meaningful in social capital formation and impact. For example, Pelling and High (2005) suggest that “…urban communities tend to have strong bridging but weaker bonding capital, whereas rural communities more typically have strong bonding but weaker bridging capital”, (p. 313).

**Table 1:** A framework for analyzing bonding, bridging and linking social capital from numerous scholars including Szreter and Woolcock (2004), Kawachi et al., (2004), Almedom (2005), Newman and Dale (2007) and Aldrich and Meyer (2015)

Types of social capital	Brief definitions
(a) Bonding social capital	Trust and cooperative relationships within groups “…trusting and cooperative relations between members of a network who are similar in terms of social identity [such as family or friends],” (Kawachi et al., 2004). “… [with similar] demographic characteristics, attitudes, and available information and resources”, (Aldrich and Meyer, 2015) “…refers to trusting and cooperative relations between members of a network who see themselves as being similar,…” (Szreter and Woolcock (2004)
(b) Bridging social capital	Trust and cooperative relationships between groups “…[connections across] social groups, such as class or race. These ties are more likely to display demographic diversity and provide novel information and resources that can assist individuals in advancing in society”, (Aldrich and Meyer, 2015) “…comprises relations of respect and mutuality between people who know that they are not alike in some socio-demographic,…” (Szreter and Woolcock (2004)
(c) Linking social capital	Relationships between formal power and hierarchical structures “…connects regular citizens with those in power”, and/or “…interacting across explicit, formal or institutionalized power or authority gradients in society”, (Szreter and Woolcock)

Social capital theory suggests that increased social capital will lead to increased community resilience to disasters. The driving mechanism is hypothesized to be collective action, where increased cooperative activities that prioritize group level interests over individual interests, better enables a return to the rhythms of daily life through the provisions of needed social and economic services (Mayunga 2007; Norris et al. 2008; Bolte and Eucker 2012; Aldrich and Meyer 2015). In practice, social capital may influence what Lindell (2013) refers to within a proposed Disaster Impact Model as ‘emergency management interventions’ such as preparedness and ‘event-specific conditions’ such as improvised response and recovery. However, few studies provide in-depth qualitative case studies detailing what those interventions and conditions look like in during a disaster.

### A sustainability science approach

This research is rooted in an interdisciplinary sustainability science perspective (Jerneck et al. 2010; Lang et al. 2012), drawing on social anthropology, political science and social-ecological systems analysis. Although it is not my expertise, or the focus of the paper, the research can be closely linked to psychology. The earthquakes provide a problem oriented starting point based on the needs of local actors to respond and recover to disasters in a practical way that can rebuild and sustain their livelihoods. Understanding what worked and what created difficulties during the August 2018 earthquakes can inform how local actors prepare and respond to potential future events (e.g., COVID-19) given system constraints and conditions. From this perspective, the research questions aim to provide a combination of system, target and transformative knowledge (Regeer and Bunders 2009; Partelow and Winkler 2016). Fundamental research in the study identifies the system components, actors and events for descriptive analysis. The goals of local actors and transformative processes of change are also implicitly identified, i.e., what transformation processes work in practice for local actors given the system conditions and goals. In doing so, a social capital approach for understanding the emergence of collective action as a community-based solution to disaster risk reduction and response was taken based on prior understandings of the community and preliminary explorative communications with local actors regarding the events. Data collection aimed to understand these aspects, not only describing and analyzing the events, but also discussing how the island functions as a community and how change evolved given the context. Understanding and reflecting on researcher positionality is an essential part of sustainability science (Horcea-Milcu et al. 2019; Horlings et al. 2020), in part stemming from ethnographic social anthropology (Venkatesh 2013). My positionality is an important feature influencing the research, including my culture and values, prior assumptions (e.g., scientific training) and role as a stakeholder in the system myself (e.g., as one who benefits from having access and doing research there). In other words, by researching collective action and social capital, the degree to which I have become a part of the processes that influence their emergence and formation should be considered.

This study examines the role of social capital on community resilience following numerous severe earthquakes on Gili Trawangan, Indonesia in August 2018. The research question explored in this article is: what is the relationship between community social capital and the resilience of an earthquake response and recovery? On Gili Trawangan, the role of pre-earthquake social capital is examined in the immediate earthquake response and recovery. Second, how the response and recovery influenced post-earthquake social capital is also explored. Findings describe the emergency management interventions, improvised response and recovery processes and event-specific conditions in the minutes and hours following the earthquake, up until 3 months afterwards. The bonding, bridging, linking framework is then used to theoretically analyze findings.

## Methods

### Case description: Gili Trawangan, Indonesia

**Fig. 1:**
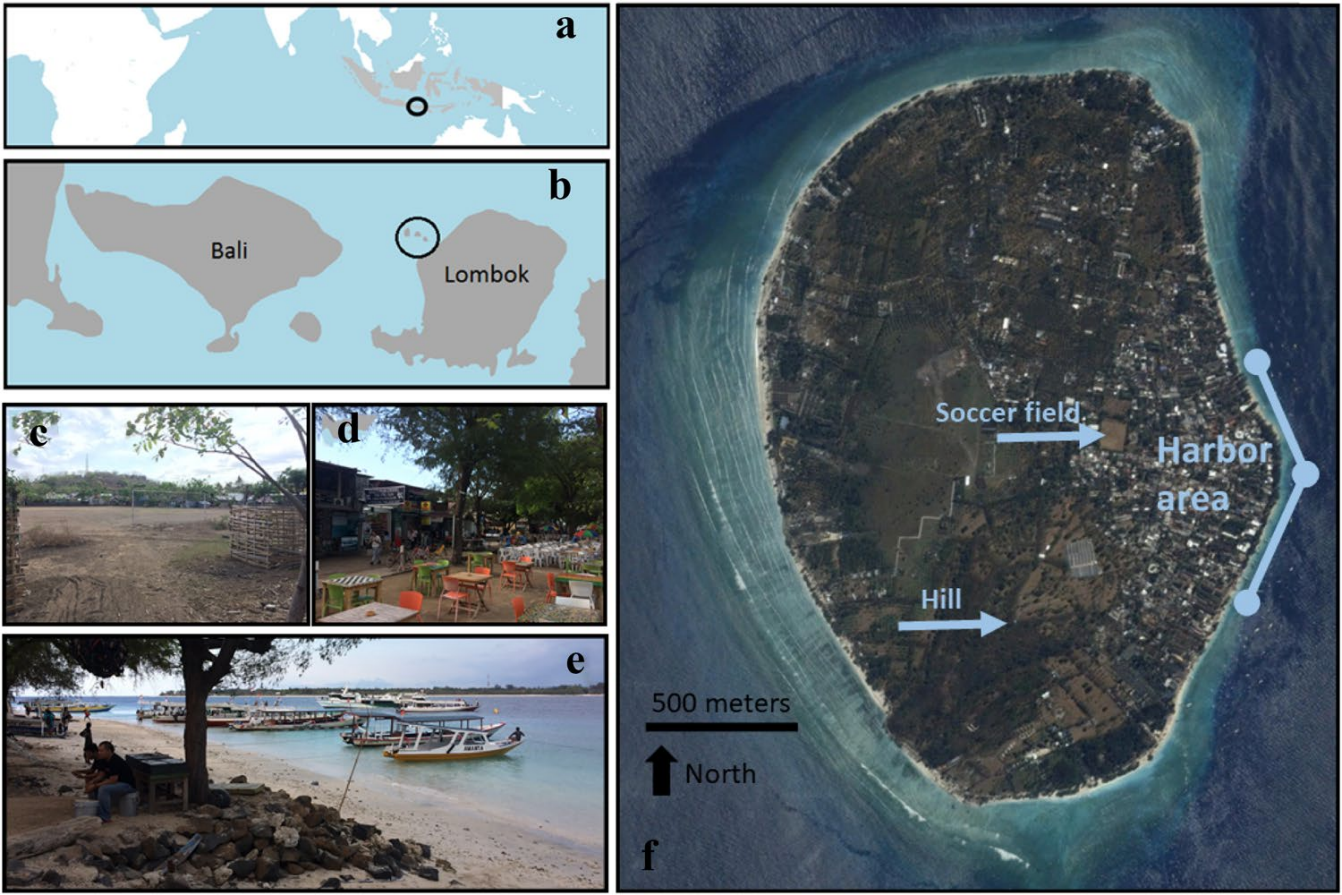
**a** Location of Lombok within Indonesia (highlighted in gray). **b** Location of the Gili Islands (circled) in relation to Lombok. **c** Looking south across the soccer field towards the hill on Gili Trawangan. **d** Boardwalk in the eastern harbor area. **e** Beach front in the eastern harbor area. **f** Satellite photo of Gili Trawangan (Google Earth)

Gili Trawangan is the largest (~ 4 km^2^) of the three Gili Islands off Lombok, Indonesia (Fig. 1). The island provides the Lombok economy substantial economic development opportunities beyond fishing and agriculture, with relatively high wages for locals who can speak basic English. An estimated 750 businesses, 2500 permanent residents and up to one million tourists per year utilize the island (Partelow and Nelson 2018). SCUBA diving and tourism have steadily built the economy since the 1990s (Satria et al. 2006; Charlie et al. 2012; Graci 2013; Hampton and Jeyacheya 2015). Around 43 diving centers exist on the three Gili Islands, along with many hotels, bars and restaurants (Partelow and Nelson 2018). The island is easily accessible, 2 h from Bali or twenty minutes from Lombok with one of more than ten boat companies. All consumable supplies on the island are imported, and only some recyclable waste is exported. Nearly, all public infrastructure and services have been self-organized by businesses, residents and heads of the island including schools, waste management, sewage, on-island transportation, environmental conservation, safety, medical services and social-political organization (Willmott and Graci 2012; Partelow and Nelson 2018; Nelson et al. 2019).

The island is a microcosm of self-organized collective action among SCUBA industry actors. Participation by some local residents, restaurants and accommodation businesses have followed. Foreign business owners, local Indonesian staff (commuting daily from Lombok), young SCUBA dive professionals and resident locals combine with international tourists. However, the island’s popularity has increased the pace and scale of governance challenges, requiring continuous adaptation and collective action. An environmental non-governmental organization (NGO) was self-organized by the SCUBA industry, the Gili EcoTrust (https://giliecotrust.com/), along with Gili Indah Dive Association (GIDA) (https://giliindahdivealliance.com/) to maintain safety standards and cooperation. An economic price agreement for diving as well as fundraising mechanisms for waste management are also ongoing efforts (Graci 2008; Partelow and Nelson 2018). However, tourism development may be best explained as “too quick to deal with the problems it creates”, (local Indonesian leader).

The island has economic and political decision making hierarchies represented formally through organizations and associations, as well as informally through friendship networks. Many development decisions have been made behind the scenes. ‘Life on the island’ is a daily orchestration of trust, reciprocity and information sharing. Overtime a ‘family tree’ of SCUBA business owners and staff has formed, mirroring informal social networks (Partelow and Nelson 2018). Relationships between non-Indonesian owners and staff, and local Indonesian workers, have been cited as both not only very positive but also difficult culturally, due to different work and communication expectations. This study estimates that dive centers on the Gili Islands employ more than 850 local Indonesians living in Lombok.

### Data collection

“Social capital is one such complex issue that benefits from the coherent integration of qualitative and quantitative approaches”, (p. 2) (Jones and Woolcock 2007). Data collection and analysis followed the 32-item checklist from Tong et al. (2007) (Appendix Table S3 in Supplementary material). Multiple types of data collection methods were used including qualitative interview data through purposive sampling, survey data through non-stratified random sampling and participant field observations. Qualitative interview data were collected throughout November 2018 with key informants (*n* = 44), using both open-ended and semi-structured interview questions (Table 2). Key informants were selected through their knowledge or first-hand experience of the earthquake response and recovery processes, as well as individuals actively involved in the island’s social, environmental and political issues. Numerous shorter exploratory interviews were conducted with business owners and local staff to help identify further key informants and cross-check events. Approximately half of the key informants were identified prior to the beginning of data collection through previous contacts from prior research, the remaining identified through snowball sampling during the fieldwork phase. Due to the international tourism influence, all interviewees spoke sufficient English to conduct interviews.

**Table 2:** The number of key informant interviews conducted with different stakeholder groups for this study

Key informant interviews		Stakeholder group (with in-text abbreviation)	Average interview length (min)	Length of all interviews
Open-ended	Semi-structured			
3	5	Foreign-owned businesses (FB)	44	5 h, 55 min
4	2	Indonesian businesses (IB)	26	2 h, 40 min
–	1	Local Indonesian leaders (LIL)	60	1 h
5	5	Local NGOs (NGO)	41	6 h, 55 min
9	9	Foreign-owned SCUBA businesses (FSB)	33	9 h, 55 min
–	1	Indonesia SCUBA businesses (ISB)	45	45 min
21	23	- Total -	37	27 h and 16 min

Semi-structured questions were guided by the six dimensions of social capital including (1) groups and networks, (2) trust and solidarity, (3) collective action and cooperation, (4) information and communication, (5) social cohesion and inclusion, and (6) empowerment and political action (Jones and Woolcock 2007) (Appendix Table S1 in Supplementary material). Interview questions followed a diagnostic approach, starting with broadly relevant questions and then continually refining them based on the information obtained (Ostrom 2007; Cox 2011). Data were triangulated between interviewees to confirm events, actions and other responses. This was done to avoid in-group biases through sampling via multiple entry points into an actor group. The sampling strategy and number of interviews in the study aimed to achieve data saturation (Fusch and Ness 2015). Qualitative data from each interview were digitally transcribed from detailed notes taken throughout and after each interview due to preferences to not be recorded and requests to remain anonymous by not recording. Although not all interviewees preferred this, for methodological consistency, the same approach was taken with all interviewees. Transcriptions were made directly after each interview.

Two quantitative surveys were conducted between November 2018 and January 2019. The first survey purposively sampled the entire population of SCUBA business owners or managers on Gili Trawangan (*n* = 22) (Appendix Table S2 in Supplementary material). This survey generated descriptive data on each SCUBA center as well as Likert responses to questions on perceptions of how the earthquake affected cooperation and tourism to triangulate with qualitative data. A second survey was conducted on a non-stratified random sample of tourists who had been SCUBA diving on the Gili Islands during their current vacation (*n* = 389) regarding perceptions of travel and safety related to the earthquake. Tourists were sampled in the harbor area before leaving the island, from each of the eleven boat companies randomly on different days and times.

### Data analysis

A content analysis (Stemler 2001) of qualitative interview data was split into two parts, with the overall aim to “both value and incorporate experiential knowledge into the analysis of development successes and failures”, (Dudwick et al. 2006). The qualitative data coding program MaxQDA was used to organize the qualitative data, import the bonding, bridging and linking coding framework (and constituent sub-codes). Text was coded and sorted in the program. First, a descriptive chronology of the events was generated for the post-earthquake period. Interviewees remain anonymous and direct quotes are labelled to the stakeholder group of the individual’s affiliation (Table 2). Second, content was coded into bonding, bridging, linking ties (Table 1) as an overarching deductive framework for the analysis. Within this framework, inductively derived subcodes were generated to sort the specific experiences, reflections, actions and events expressed by interviewees. Quantitative data from both surveys were formatted and analyzed descriptively using the statistical program R “base” package (R Core Team 2018). Descriptions of the samples are in Appendix Table S2 in Supplementary material.

## Results

After the earthquake on August 5th, 2018 earthquake (~ 7:45 pm), many fled towards the island’s only hill with fears of a tsunami, while others made their way to the eastern harbor, where a majority of the businesses and people are usually located. The island’s typical ~ 10,000 tourists during a high season week was substantially lower (less than 5000), due to high seas blocking public speed boat traffic from Bali for 2 weeks prior. This also blocked aid response and evacuations, forcing evacuations through Lombok. Mainland North Lombok faced far more devastation, the Indonesian Ministry of Foreign Affairs reported 556 deaths, more than 1000 injured and over 417,000 internally displaced people.^2^

Numerous response and recovery processes occurred following the earthquake, chronologically outlined below. Event descriptions (Sects. 3.1, 3.2, 3.3) (Table 3) are juxtaposed with anonymized individual interview quotes, labelled by stakeholder group (Table 2). Section 3.4 synthesizes findings within the bonding, bridging, linking social capital framework, and provides interview quotes on the role of social capital in the response and recovery.

**Table 3:** Chronological description of events following the earthquake in the minutes, hours, days and weeks following

Timeline	Event
0 min	6.9 earthquake off the coast of North Lombok, August 5th, 2018 at ~ 7.45 pm local time
15 min	Cell Phone service and electricity goes out Panic of pending tsunami; many rush towards hillMany others start walking towards eastern shore/ harbor area
1 h	Most people now on hill, soccer field or harbor area beach Many foreign staff/owners went home to check houses, get clothes/ supplies
3 h	Three improvised triage/ medical areas started; largest near harbor Dive professionals and volunteers assisting critically wounded Some foreign residents helping people with water, food, calming people down Some electricity generators now working
First night	Medical shifts of those volunteering to help injured Improvised medical book (at harbor) to log medications, vital signs and status of injured in systematic way Most sleep outside on the beach or soccer field (1000 s of people) or stay up all night on the hill with panicked group (100 s of people)
Morning after and day 1	First public and private boat companies begin to leave the island Many people panicking (mostly tourists) to get off the island High swells block boats to Bali; everyone leaving must go to Lombok Some dive center boats take staff and injured to Lombok Looting begins while island is still chaotic with most trying to leave Search for medical supplies around island by volunteers helping Multiple businesses running generators for everyone, to charge phones, etc Free food and drinks being given by multiple businesses
Day 3	Indonesian Minister of Foreign Affairs comes to island stating, “If you don't leave, you’re on your own”. They cannot help foreigners, so all should leave Nearly everyone evacuated off the island Rumors of organized looting, violence and assault
Day 4—21	Groups of foreign residents (~ 80–100) stay to assist with clean-up/rebuilding Numerous small groups around island, but one large group (~ 40–50) in the harbor area to assist with most recovery efforts Groups formed to do needed tasks including clear all kitchens and businesses of rotten food to avoid disease (and enable quick returns back), to mark/assess damaged buildings, to help animals (cats and horses), to fix water towers and electricity lines, to help injured locals who stayed on hill, to cook food for all, and to scavenge supplies, among other tasks Check-in and our safety board developed and team system (pairs) organized for always going out to do work Groups on Bali set up online fundraising efforts and use money to buy aid supplies for communities of employees on North Lombok; assisted by foreign established NGOs and businesses linked to Gili Trawangan
August 19th, 2018	Two more large earthquakes Minor damage; no additional deaths or critically injured on Gili Trawangan
September 1st, 2018	Island officially opens for tourism again Many businesses still closed
Following 3 months	Businesses attempt to spread message encouraging tourists to come to the island, as the best way to help locals and the area recover Slow return of local Indonesian employees to work on Gili Trawangan, North Lombok recovery much slower Psychological trauma stated as influential for Indonesian employees not returning to work; foreign residents reflect on personal and the island’s future

### Collective action in the hours after

Those injured and requiring immediate medical attention became first priority, and within the first hour, 100 s gathered in the harbor area with dozens seeking first aid and critical care. A long-term SCUBA business owner stated, “the response after the earthquake made it seem like we had a plan, even though we didn't” (FSB). Many intuitively provided reactive assistance and gathered in groups near one of the main social hubs of the island, one of the largest and most centrally located SCUBA centers with a high degree of social connectivity (Partelow and Nelson, 2018). This quickly became a triage center and gathering place for panicked tourists, locals, and critically wounded. Mandatory first responder training from the community of SCUBA instructors and dive masters was valuable for urgent care and saved lives. Despite no emergency plan, leadership among many individuals to create some order, delegate tasks and assess who can help with the skills they have (e.g., medical care, calming people, finding supplies) defined a majority of the first-hand accounts.

**Fig. 2:**
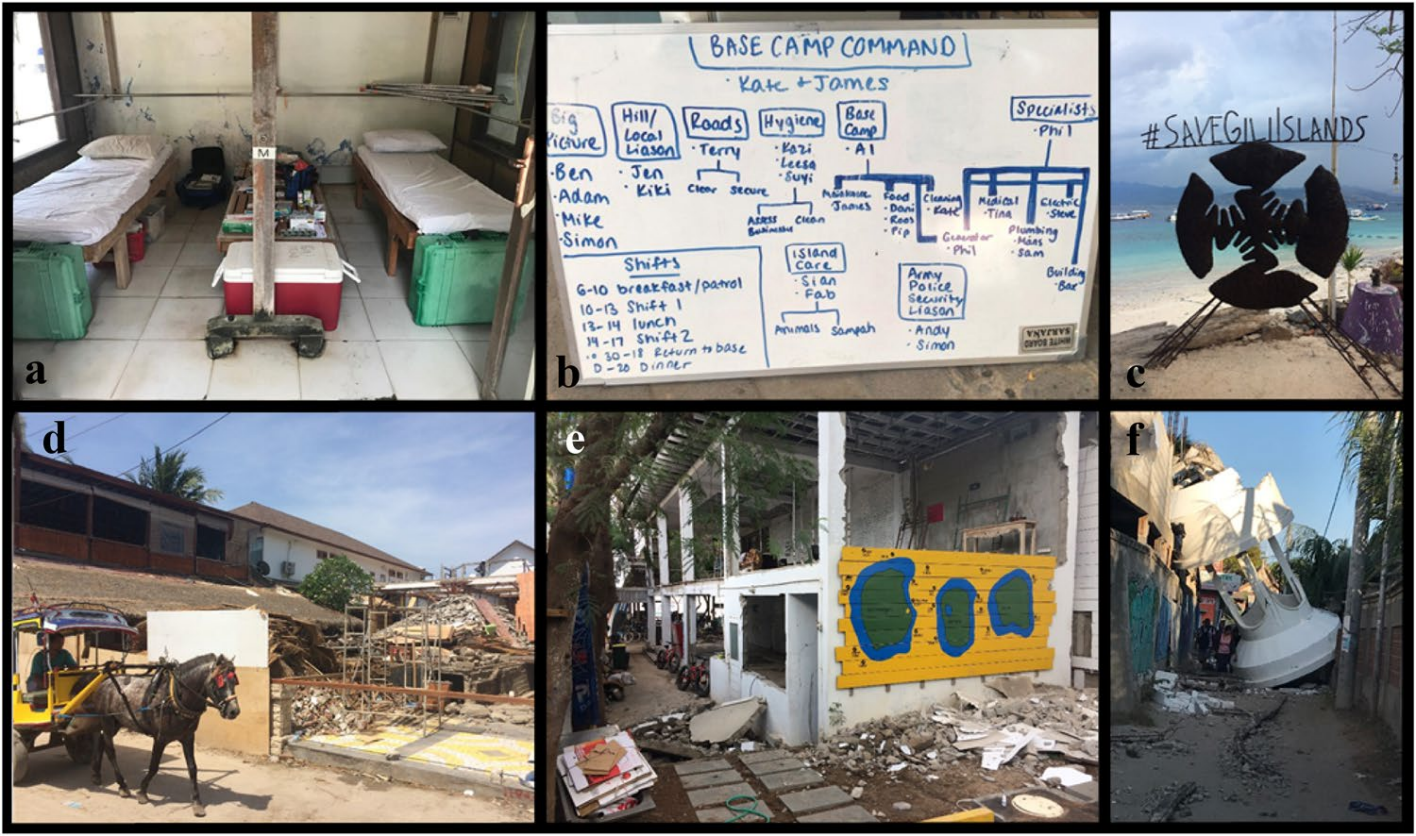
**a** Diving equipment storage room converted to medical area the night of the earthquake. **b** Organizational board for the group who stayed on Gili Trawangan in the harbor area, showing the daily schedule and who (in pairs) will do what tasks during the day to help the island recover. **c** Artwork made to promote the recovery of the islands in the main harbor area. **d** Destroyed SCUBA center in the main harbor area; a horse cart, the only transportation option besides bicycle on the island. **e** A destroyed SCUBA center. **f** Fallen tower of the local mosque onto a main road

A rotation system was initiated for monitoring and caring for the injured throughout the first night in the harbor area. From one first responder, “you don’t know what you're capable of until you are put into that situation”, (FSB). An impromptu logbook describing the patients, marking each with a number on their forehead, their injuries, vital signs and medications or care given was organized (Fig. 2). Lounge chairs and equipment storage rooms provided resting and care areas for injured (Fig. 2). Deceased were brought to the beach in the harbor area and prepared for transport off the island. Other foreign residents helped calm tourists on the beach, find food and water, scavenged medical supplies or helped those without cellular phones or service make calls.

Away from the eastern harbor area, 100 s spent the night on the island’s only hill fearing a tsunami (Fig. 2), including many terrified Indonesian island residents with reports of continuous shouting, crying and praying. Many were injured, and those who could, provided medical assistance. Another large group spent the night on the soccer field, one of the only large areas besides the beaches clear from rubble. Fortunately, no tsunami came, although it would have been possible depending on the earthquake size and location (Mueck et al. 2013).

Findings suggest that the pre-existing social capital (primarily in reference to bonding ties among FSB and FB) created a mentality of collective action and agency in the island’s community. This enabled ‘social preparedness’, making the immediate response and later rebuilding efforts more resilient. Although “everyone reacts differently” (FSB), the island’s history of self-organization played a role in how the community responded.

### Collective action in the days and weeks after

The morning after, private boats from SCUBA centers and public boats began transporting the injured, dead and local Indonesian staff (numerous interviewees estimated ~ 90% of Indonesian staff on Gili Trawangan live on Lombok), back to Lombok for evacuation. Due to halted public boat services from high seas, boats leaving the island were mostly self-organized by individual businesses, causing a general rush of tourists to the beaches, promptly trying to leave. “Everyone was trying to get off the island…but those who were helping the relief effort created a human chain from the harbor area to the boats, to block tourists and get the injured and dead off the island first”, (FSB). It was mentioned that some tourists had the mentality that they were more important than locals in terms of who should get off the island first, although it was clear that locals need to get back to their families in need on Lombok, and all tourists were simply on vacation in no immediate danger. Many foreign residents warned against immediate evacuation, as the island was likely the safest place to be if you cannot get to Bali. Numerous accounts of looting were reported, done by both foreign tourists and local Indonesians, especially alcohol and other goods from small shops, although many businesses were giving food and drinks away for free.

During the first 2 days, most local residents assessed household and business damage, also helping friends and staff. Many attempted to make generators, food, accommodations and water available for free. Rumors of looting and assault were strongly influential in decisions to leave the island or not, and although one or two isolated incidents occurred, this added overall safety concerns. On day 3, the Indonesian Ministry of Foreign Affairs gave a speech at the eastern harbor to remaining foreigners, saying in the words on one attendee, “if you don’t leave, you’re on your own”, as the last of the public boat services were being offered. Government capacities were being directed to their own citizens on Lombok, and they could not prioritize the safety and needs of foreign residents. Most remaining foreign residents, including most of the women and children, took the warning and were advised to leave on the last boat. However, many chose to stay, stating they “could not leave” (FSB), and that someone needed to look after the businesses and begin the recovery process, as it was clear that it would not get done unless they did it themselves. Summarized by a local SCUBA owner, “spread of information was [minimal] from the government, so this leads to self-organization”, (ISB).

#### Recovery on the island

An estimated 80–100 people, at least three separate groups of foreign permanent residents (defined by where they stayed and who they interacted with during the closure), and some local Indonesians remained on the island between August 10 and September 1, 2018. The largest of about 30–40 foreign residents all stayed at a dive center in the eastern harbor area (same as triage center), a “good location for seeing what boats and supplies were coming on and off the island”. The second small group, after rumors of looting and assault by locals from Lombok, fortified themselves on the northern part of the island. A third group of hotel owners and managers remained on the island’s northwest, but kept mostly to themselves, along with local resident Indonesians living permanently on Gili Trawangan.

The larger eastern harbor group initiated most of the collective actions, assessing and prioritizing tasks to get the island back in order. “We organized people into their different skills to do different tasks around the island. Everyone had different skill sets… [from previous careers],” (FSB). Tasks included clearing debris from roads and beaches, acquiring excavators to be shipped from Lombok, assessing damaged buildings and marking them, clearing out the kitchens and organic waste from all businesses to avoid rot and disease, fixing generators and water towers, assisting the locals who remained on the hill for weeks (still in fear of a tsunami), attending to the horses and cats running loose or set free on the island by those who left (also done by second group on the north of the island) and cooking for everyone (Fig. 2). Daily groups of at least two, for safety, were self-organized to complete tasks (Fig. 2). A curfew of ‘back before dark’ was self-imposed for safety. During these 3 weeks, a substantial amount of collective work was done, allowing the island to officially reopen on September 1, 2018 with functional access to public areas, infrastructure and businesses.

Although it is clear that “the cooperation of western businesses on issues keeps things together on the island, and working”, (FSB), the collective actions following the earthquake were not without contention. For example, how the animals should be dealt with, because “saving the horses is saving people's [i.e., local horse cart drivers] livelihoods” (FSB), similar to reopening businesses for staff employment. “Older shops on the island had to keep together”, (FB), to resolve many of the immediate problems without conflict to get the island functioning again (e.g., who will bear the costs of organizing and financing the collective work). Motivations to quickly reopen businesses soon after the earthquake received critique. Reopening was a goal for many to support local staff salaries, but criticized as self-interested by some. However, nearly all agreed that rebuilding tourism on Gili Trawangan is the best possible way of supporting Lombok and local Indonesia staff. “Lombok [and Gili Trawangan] will recover faster because of tourism, compared to Palu^3^” (LSB). Stable income and a return to normalcy was repeatedly stated by businesses as critical for local Indonesian staff and the economy. As stated by one politically influential local Indonesian, “[For local people] the Gilis are a diamond in the middle of the jungle”. They provide relatively high earning and stable jobs for “a large percentage of North Lombok” (FSB) or “thousands of people” (LIL) who otherwise have few opportunities besides agricultural jobs or fishing, and that “most villages [in Lombok] have a connection to the Gilis”, (FSB). Gili Trawangan can be seen as a “lifeline” (LIL) for many locals, but only if tourism is sustained.

Motivations for collective action varied, but all with the common themes of agency in being able to contribute to rebuilding community identity. This identity was often referenced to the island’s history and need for cooperation. “When back to opening [for tourism] again, managers were working together to help each other get started again”, (FSB), although their businesses are inherently competing with each other. Similarly, “loyalties were tested after the earthquake. The people who were meant to stay, stayed, the people who were meant to leave, all left immediately after and won’t come back”, (FSB). Since the 1990s, there has been a sentiment that self-sufficiency has led to a proactive sense of agency among many. “If you wait for the government it takes too long”, (LIL), referring to the necessity for quick recovery processes.

#### Aid from off the island

“Gili [Trawangan] is a small island with a big network”, stated by the owner of a global SCUBA certification organization operating in Indonesia for more than 20 years. The majority of foreign permanent residents who did not stay went to Bali. Many self-organized into groups to gather donations for disaster aid supplies. Bali groups were also self-organized by individuals taking informal leadership roles (i.e., setting goals, understanding the useful skillsets each person there had, and creating an action plan). This include setting up GoFundMe pages (linked to the individual SCUBA centers) using social media networks to reach tourists who have visited the Gili Islands from around the world to bring global attention to the relief cause. They developed the social media hashtags #LombokStrong and #GiliStrong, and many made t-shirts to support the relief and rebuilding efforts. More than 15 SCUBA businesses raised thousands to tens-of-thousands of dollars (USD) each to purchase food, water, blankets, temporary shelters, clothing, medical and other supplies in Bali for shipment to villages of local staff in North Lombok. Work by those on Bali mirrored a relief organization, but it popped-up in a matter of days, explained a foreign SCUBA professional, “[anonymous] helped to organize the skills that we all had. He had everyone write down the skills that they have from previous experience. Then, he stayed up all night sorting people into groups, into teams, to coordinate a relief effort…each group/team had an objective. Social media team did a really good job at contacting the international news organizations. We had people from all the different countries of the foreigners who were there, doing interviews and the countries of major tourism visitors. They did a great job. This helped to get more donations as well. [The] cultural diversity of the tourists and of the staff working here in Gilis helped the relief effort”, (FSB).

Donations were aggregated and used to purchase aid supplies, and shipped to Lombok in large containers. “The relief effort was totally self-organized, organized with the police in Bali. They used a government/police boat to transport the supplies to Lombok”, (FSB). Knowledge of what was needed and where supplies would be most useful was organized through connections with existing non-governmental organizations on Lombok affiliated with Gili Trawangan businesses, such as the Pituq Community Foundation. Tourism provided a global network of financial resources. “The international nature of tourists, the high international turnover of Gili [Trawangan] helped to raise money, and made the relief effort faster for this reason because they could get needed supplies to North Lombok and then get back [local staff living on Lombok] to Gili to start the economy again”, (FSB).

One challenge mentioned by numerous interviewees, emphasizing the need for and usefulness of self-organized supply chains and aid relief, was corruption issues in the delivery of government aid through traditional supply chains. Foreign or government aid could easily be rerouted to certain areas of political or personal interest, to leverage economic or political gains for individuals with power positions along the supply chain. This was reported in Lombok, that a substantial amount of aid supplies were diverted from where they were needed most, stagnating the recovery efforts, making the self-organized and community-based collective action even more impactful as a means of building local resilience.

The social networks of Gili Trawangan extend beyond the island. For example, one of the world’s largest SCUBA certification agencies agreed to donate all SCUBA certification profits coming from the island for 3 months. Each SCUBA center could decide where the donations would go, due to “trusted relationships” (FSB) and personal connections with Gili Trawangan’s SCUBA businesses. The Gili Islands provide “approximately 30–34% of the SCUBA certifications in Indonesia”, (FSB). Similarly, a public fast boat company that is informally ‘in the social network’ delivered supplies during the closure at no cost. Similarly, to clear debris “[an Indonesian business owner] shipped two diggers directly to Gili [Trawangan] after the earthquake because of connections to government in Lombok”, (LIL).

For many local Indonesians, the Gili Islands are a “lifeline”, (LIL), but the relationship is interdependent. Businesses would not be able to operate without local labor, either economically or legally.^4^ Nearly all foreign owned businesses recognize local Indonesians as the foundation for a functional tourism economy. This was reflected in recovery efforts, as the focus of most businesses was to get aid to communities of their staff in Lombok. This helped in two ways. It showed local staff that businesses support them, not only as employees, but as integral parts of the Gili community and as extended family. Second, it gave practical aid catalyzing recovery so they could come back to work. Many businesses continued to pay local Indonesian staff at the minimum wage rate (~ 30 USD per week) throughout the closure period. However, “no [foreign] staff got wage during the closure period” (FSB).

### Long-term recovery and reflection

It is evident that collective actions allowed Gili Trawangan to officially open for tourism on September 1st, 2018. Getting back to a new ‘life on the island’ was recognized as a long-term process, but sentiments of interviewees reflect that it will be positive. Interviewees reflected on the substantial tragedy, but noted many positives, such that “everybody realized that Gili [Trawangan] is one community after the earthquakes”, (NGO) and that “we are all in the same boat”. In reflection on the island self-organized the recovery, one respondent commented, “I don’t think that the community will forget that we helped each other”, and that you can “go through disaster but come out stronger afterwards”, (FSB). A common sentiment was that “before the earthquake, we knew each other, but now it's more connected”, (FSB), and that “the island is now close together due to the disaster. We became really close. We knew and worked with each other before, but know we are really close. Helping each other for the relief effort…This experience brought us together”, (FSB).

Some had more critical reflections. As stated by one politically influential foreign resident, the “biggest lesson from the earthquake is that you can classify people, to see who is invested in the island”, with a long term sustainable view. “Many on the island saw the same things, but reacted to them in different ways. Some left the island forever, and some were motivated to come back”, (FSB). However, some business owners and staff (foreign and local) simply did not come back, or took individualistic actions in rebuilding, dealing with staff or contributing financially to collective recovery efforts. Many stated it became apparent what recent businesses cut corners on building costs. The island’s growing popularity has brought new foreign investors not integrated into the island’s social networks. Some are perceived as trying to maximize short term gains with unsafe and non-environmentally friendly building materials and methods, many of which collapsed and left behind non-recyclable material.

Self-organized collective action as a means of informal governance (i.e., social organization and decision-making) also has downsides. “Self-organization helped on one hand; partly solving problems, but also creates new ones”, (FSB). This included a lack of clear guidelines and rules, defined leadership roles or clear mechanisms for contributing to group financial costs. Established communication channels helped mediate the negative aspects, and certainly aided the response and recovery. “Communication about the island after the earthquake was going through GIDA, their WhatsApp^5^ group. GIDA infrastructure was already established, so they used this to hear what everyone was doing”, (LSB). New WhatsApp groups were started among Bali and Gili groups. However, communication was often difficult between businesses and local staff, who understandably left for Lombok after the earthquake, but never came back or communicated when they (staff) would return to work. Reflections differed on business-staff relationships, with one SCUBA owner stating that “the biggest thing that occurred from the earthquake was the better relationship between the foreigners and the locals [living on Gili Trawangan],” (FSB). Another stating that it is “hard to say that the relationship with locals got better. Some got better, some got worse”, (FSB). Referring to those who stayed during the closure, “we did the collective work for the whole island. The locals were helping as well. We worked together and it brought us closer together with the locals. They would know my name because I am the owner of [a large SCUBA center], but I didn’t know their name. Now I know many of them, and we are closer than before. We would cook them food [during the closure period]. This made the relationships with the locals better, we trust each other”, (FSB).

A common reflection was that religion played a role in how locals Indonesians responded, and why many locals faced substantial psychological trauma, as stated by their employers. “Local people [Indonesians] fear the earthquake, because god controls nature. This is why the earthquake has created so much trauma…”, stated a local politically influential Indonesian (LIL). However, psychological trauma affected most people who experienced the earthquake. Most interviewees mentioned that they suffered some degree of post-traumatic stress disorder (PTSD) (in their own words, as this study did not ask these questions intentionally or aim to diagnose any medical conditions). Although this study did not focus specifically on psychological impacts, it was repeatedly mentioned that local Indonesians have suffered the most in this regard, with numerous interviewees, including Indonesians, stating what could be summarized as a collective trauma about how to deal with and recover as individuals and as a community.

Part of the long term recovery process will be psychological. As one business owner (FB) stated, “it is a hard relationship with locals [after the earthquake] because ‘logic’ [in reference to taking ownership and action in the recovery] can’t compete with ‘fear’”. While many foreign business owners wanted to bring staff back to work and pay them, providing them income and a psychological break from immersion within a disaster mentality, many struggled to empower their staff to work and get back to normal. Among foreigners, social bonds were beneficial and relied upon throughout the event, and even created by going through it together. At least three interviewees got tattoos remembering some aspect of the event, and many stated it changed the way they think about the island and personal development. The following reflection represents a common theme. “I have a friend [who lived and worked on Gili Trawangan before the earthquake]. Her experience after the earthquake, made her immediately leave and never come back. She is now seeing a psychologist…and often texting me why I don’t leave and why I continue to stay. She doesn't understand. I need to see the island recover, I am close with the staff and need to see it recover. I was planning to leave next year, but now I will stay”, (FSB).

Among SCUBA businesses, survey data suggest that there was near consensus that the earthquake generally brought businesses together to help each other, and that it did not create more conflict (Table 4). Similarly, there was positive consensus on cooperative trust and reciprocity, that each business trusts that others would help in an emergency and that they would help in return. Nearly all were optimistic that the island will return to normal, and that the earthquake provided an opportunity to implement needed change.

**Table 4:** Responses from SCUBA center managers/owners regarding the impact of the earthquake

Statement	Strongly agree	Agree	Disagree	Strongly disagree	N.A
The earthquake changed the relationships between SCUBA businesses in a negative way	0	5	5	12	–
The island will be worse off, overall, due to the earthquake	1	6	10	3	2
I trust that other SCUBA shops on the island will help me if there is another disaster	12	10	0	0	
The earthquake generally brought businesses together to help each other	10	11	1	0	–
SCUBA tourism on the island will eventually go back to the way it was before the earthquake	6	14	2	0	–
After the earthquake, businesses on the island all helped each other	2	16	3	1	–
The earthquake provides an opportunity to implement needed change on the island	5	14	2	1	–
I would help other businesses, if needed, during a disaster	13	9	0	0	–
The earthquake generally created more conflict between SCUBA businesses on the island	0	2	11	9	–

Tourists visiting 3 months after the earthquake were asked questions regarding the earthquake and their travels (Table 5). More than 87% of tourists had heard about the earthquakes before they traveled, with only 25.7% stating they had concerns for their safety, although 71.2% were first time travelers from all over the world (Appendix Table S2 in Supplementary material), although the latter statistic is biased, because it did not sample those who changed their plans and did not come, and only sampled SCUBA divers.

**Table 5:** Tourist responses related to the earthquake, safety and traveling

Statement	Yes	No	N.A
I was aware of the severe earthquakes in August before I arrived on Gili Trawangan	340 (87.4%)	46 (11.8%)	3 (0.07%)
I changed my travel plans due to these earthquakes	10 (2.5%)	376 (96.6%)	3 (0.07%)
I was concerned about my safety before this trip	100 (25.7%)	285 (73.2%)	4 (0.1%)
This trip was my first trip to the Gili Islands	277 (71.2%)	108 (27.7%)	4 (0.1%)

### Bonding, bridging and linking social capital on Gili Trawangan

Bonding ties are assessed here as the strongest form of social capital on Gili Trawangan (Table 6). Local social and political knowledge within the FSB network, as well as their familiarity with regular communication and dealing with collective action problems was a dominant theme when interviewees explained the reason why the recovery was rapid and cooperative. The island’s pre-existing networks are interpreted as bonding ties. “It would not be possible to start a business on the island, or very difficult, without having informal network connections with existing businesses…Having this network helps make everything easier and possible”, (FB). A 30 year professional in the SCUBA certification industry stated that “SCUBA shops on Gili Trawangan work together, unique for the area”, and that “cooperation keeps the quality of the diving certifications high on the island”, (FB). Furthermore, that “[this individual] has never seen so many shops that are not trying to one-up each other…in Southeast Asia…[or] worldwide”. In reference to established networks for communications, “GIDA made people prepared mentally. People were used to calling each other and being in contact before the earthquake, (FSB)”.

**Table 6:** Synthesized results coded and subcoded into the social capital framework of bonding, bridging and linking ties

Types of social capital	Subcodes	Synthesized examples
Bonding	Networks^a^	- FSB economic network; enhanced trust and cooperation - FSB and FB social network enhanced, shown in this study. Other groups likely as well
	Cognitive^b^	- Shared experience - Mentality of self-organization and agency maintained - Sense of responsibility to help island/ see it recover revealed - Collective belief you will help others, and others help you - Collective belief earthquake is an opportunity for positive change - Earthquake did not increase conflict; rather increased bonding
	Activities and actions^c^	- Many individuals took leadership roles and/or risk - Many individuals (and businesses) donated time and/ or money - working together on the island for common goal for multiple weeks - Use of existing communication channels; Sharing needed resources - Continued work for the recovery/ island despite no pay
Bridging	Networks^d^	- FSB connecting with local Indonesians/ staff - FB connecting with unacquainted FSB - Foreign staff, local staff, FB and FSB building new relationships - NGOs, local staff, FB and FSB connected via relief aid efforts
	Cognitive^e^	- Shared experience of earthquake - Recognition of social and economic interdependencies among all groups - Sense of community strengthened across groups
	Activities and actions	- Self-organized relief in Bali for Lombok; working together for a common goal - Procurement of transportation and supplies - Local Indonesians and FSB fixing island together during closure - Self-organization of skills: medical, technical, emotional, building, military, leadership
Linking	Networks^f^	- Tourists connecting with local Indonesians via FSB networks - International and regional businesses connecting to FB/ FSB and local staff on Lombok
	Activities and actions	- FSB/ FB connected tourists to local Indonesians through establishing relief aid networks - Self-organized financial and material aid helped staff on Lombok return to normal - NGOs (Pituq; Gili EcoTrust) shifted attention to aid relief - International and regional businesses contributed money (donations from SCUBA certification organizations) and resources (i.e., transportation and material supplies) due to personal connections with local businesses

Stakeholder group codes: foreign-owned businesses (FB), Indonesian businesses (IB), local Indonesian leaders (LIL), local NGOs (NGO), foreign-owned SCUBA businesses (FSB), Indonesia SCUBA businesses (ISB)

^a^Networks (bonding): social networks of shared norms and reciprocity

^b^Cognitive (bonding): “social control/ efficacy; shared values; mutual trust and norms of reciprocity”, (Almedom 2005)

^c^Activities and actions (bonding; bridging; linking): tangible events influencing response and recovery

^d^Networks (bridging): “access to public goods and services, amenities”, (Almedom 2005)

^e^Cognitive (bridging): “participation; sense of belonging; decision-making capacity”, (Almedom 2005)

^f^Networks (linking): networks across power and institutional hierarchies

Cognitively, bonding ties were enhanced through shared experience, a sense of collective responsibility and a mentality of problem-solving that arose in many individuals in the FSB, FB, LIL and NGO communities. Motivated by the actions of others and strong community identity, many contributed to doing the necessary group work. Quantitative results confirm qualitative understanding that the earthquake did not increase conflict, but that there were many positive aspects including opportunity for changing personal trajectories or the island’s, and increased bonding between most groups. Among those who stayed on the island during the closure, “…we made the rule that by sunset everyone had to be back…for safety. Then, we would eat and have drinks and try to have fun together. We drank too much, it was fun. We still had cold beers”, (FSB). In one of the Bali aid groups, “this was ultimately a bonding experience for many people. A good example of the Gili community. The existing social network was a reason for people coming together during the relief effort in Bali”, (FB).

Bridging ties are important on Gili Trawangan, they represent interdependencies between the island’s different groups. Bridging ties existed before the earthquake, and findings suggest that bridging ties have increased after. “The idea that the social networks on the island made it more resilient in the recovery is totally true”, (FSB). Bridging ties were well established before the earthquake, making them useful for relief aid. “The social networks on the island and the mentality of self-organization, made the island recovery faster”, (IB), and that “the social network of the Gilis helped speed up the recovery effort”, (FB). Shared activities during the response and recovery broadened connections between groups (i.e., Indonesians and foreigners) who have trust and reciprocity based relationships, and seemed to increase cognitive recognition for the collective identity and interdependencies they all share. The “social fabric of people came together afterwards, it was stronger. I made great friendships with many I didn't know before”, (NGO). “It would be easier to join together after the earthquake and solve problems together”, (FSB). Furthermore, that “everybody realized that Gili is one community after the earthquakes”, (LIL). While bonding ties tend to focus on strengthening existing networks, bridging ties emphasize how the network expands. The earthquake itself seemed to be the catalyst for broadening the network and collective cognitive identity, which highlights the positive influence disasters can have on social capital formation (i.e., enhanced bridging ties) under the right conditions.

Linking ties played an important role, but findings suggest to a lesser degree than bonding and bridging ties. The most important being the global connections that FSB have to tourists in Europe, North America and Australia, providing an influx of aid donations and media attention. Linking ties also helped procure needed supplies locally through business and government connections via FSB and LIL. Linking ties connect people across social institutions such as class and power structures. Connecting previous Gili Trawangan tourists to local Indonesians online was an important aspect of relief aid, both not only practically in the sense of financing needed aid through online donations, but also cognitively in the sense that both business and local Indonesians now know that there is a meaningful global network willing to support recovery.

## Discussion

This study supports existing literature suggesting an iterative and positive link between social capital and community disaster resilience, manifested through collective action. Similar findings have been documented in diverse cases e.g., (Woolcock 1998; Larsen et al. 2004; Bolte and Eucker 2012; Aldrich and Meyer 2015; Sadri et al. 2017; Chan et al. 2018; Wei and Han 2018; Li and Tan 2019). Future work would benefit from exploring, not if there is a relationship, but the role of context in shaping the degree of and nuances of the relationship. How and why does social capital influence collective action across contexts differently? (see Hanna et al. 2009; Zobel and Baghersad 2019)). It is evident that social and ecological conditions, and the type of disaster, can be influential.

Many disaster contexts create spontaneous collective action problems for entire communities who need aid, but the individual incentives or capacities may be lacking to provide them. It can be assumed that communities prefer to be resilient, and to receive the benefits of collective action, and to quickly resume “the rhythms of daily life” (Aldrich and Meyer 2015). Findings above suggest that social capital likely enhances the chance of collective action occurring by lowering the social transaction costs of trust and communication. However, there may be a temporal effect. Immediate response efforts (e.g., first aid; quick organizational leadership) do not necessarily seem to be premised on prior social capital. These are more likely intuitive and reactive actions. Only once the initial chaotic and triage phase was over (in the Gili case, a few days), social capital seemed to play more of a role in motivating continued collective efforts to help rebuild as a group rather than individually interested survivalism.

Knowledge of the local context is critical for understanding what enabled network and social capital formation. On Gili Trawangan, it is evident that small group size, small biophysical size of the system, interest homogeneity, past collaboration and high group dependence on the same resource for their livelihood (i.e., coral reefs) play a role (i.e., Graci 2013; Partelow and Nelson 2018)). Without knowing this social-ecological context, it is unlikely that an analyst will see actual mechanisms through which social capital is built. These are local experiences, actions and activities unique to that place and event, including clearing debris, caring for injured, procuring relief aid and financing online, fixing infrastructure, psychological support, among others. These are the mechanisms, which we assess broadly as social capital, that enabled the return of daily life.

Do actions driven by social capital create more of it, as a positive feedback loop? Findings suggest yes. Shared activities seemed to increase cognitive bonding ties through shared experience, both negative (e.g., traumatic) and positive experiences. Previous social capital on Gili Trawangan facilitated a difficult but positive recovery, in turn further strengthening its social capital. Although much of the literature has focused on network formation e.g., see ‘The Networks View’ in Woolcock and Narayan (2000), Lin (1999) and Moody and Paxton 2009), shared experience (as cognitive bonding ties), rather than networks, seemed to influence trust, reciprocity and shared norms for cooperation as well. Shared experiences also drove bridging tie formation through experiencing that others outside your group are willing to help you and work together.

What was measured as linking ties, can be interpreted here as the use of social media (i.e., network) connections to link the victims of the earthquake to those with financial power internationally to provide aid. Linking ties helped mobilize financial resources, broadening the network of who was helping the collective action problem. Social media allowed more people to participate in the shared actions and activities for the recovery. We can observe that the social capital built through linking ties occurred through the shared activity of fundraising. This iteratively influenced cognitive bonding and bridging ties, as it showed a sense of shared values and group participation among previously unconnected actors via a shared activity.

Disasters are frequent but nearly always unique context dependent events, and the role of social capital in collective action and community resilience may differ in normal situations or in events that are less extreme. Extreme events can reveal what may be present (i.e., social capital or a lack thereof), which otherwise never gets explicitly revealed or emphasized as an important community feature, whether missing or present e.g., (Koh and Cadigan 2008; Cox and Perry 2011). For example, numerous psychology studies have examined various dimensions of increased social capital and the link to decreased post-traumatic stress disorder (PTSD) (Wind and Komproe 2012; Floresa et al. 2014) and cognitive decline (Hikichi et al. 2017; Ozaki et al. 2017) following disasters. Similarly, communities having traumatic experiences, although “painful and unwanted” often express more complex social-psychological reflections “…that which, until that point, had been largely out of their awareness”, (p. 409) (Cox and Perry 2011). On Gili Trawangan, findings above suggest that many did not reflect on the importance of the community or their sense of place and belonging on the island until the earthquake. This is reflected in statements referring to the earthquake as an opportunity for personal reflection and change, and that it brought the community closer together. The duration, magnitude or intensity of shared experience (exemplified through extreme events) seems to influence cognitive social capital formation (often without explicit physical network formation). For example, a community that experiences an event together, may increase their social capital through cognitive shared experience, although they may not have increased any physical network connections between them, they are linked through a shared experience. Further empirical work on potential subcategories for the bonding, bridging and linking social capital framework, such as those used in this study from Almedom (2005), would be useful in better understanding the specific types of events, actions and activities that drive specific types of social capital formation.

It is important to reflect on how the degree of prior self-dependency influences social capital formation and its usefulness. For example, most Gili Trawangan residents knew external aid would not come, and were well aware of potential corruption issues that may arise in formal aid distribution efforts. However, if there is a plausible expectation of external aid, such as during the 2005 Hurricane Katrina in New Orleans (Elliott et al. 2010; Hawkins and Maurer 2010) or the 2011 earthquake and tsunami in Japan, the mentality of communities might differ in how individuals conceptualize what may help them and/ or what they should do to help themselves.

We can then ask, what is the social–psychological interplay between social capital and external aid as response and recovery institutions, in cases where both are present? Also, how do community expectations of aid or self-sufficiency shape what is expected during response and recovery? This may vary at different social levels. How individuals draw on collective resources to solve their own problems may differ from how a group collectively utilizes those resource. Different groups may expect different institutions to act or be relied upon. On Gili Trawangan, the mentality of self-sufficiency in its history undoubtedly played a role in how the community responded. Context was a determining factor, but understanding how variations across context needs further research.

The severity of a disaster is important. Numerous studies have shown this to be a main indicator of post-disaster recovery outcomes (Galea et al. 2008; Zahnow et al. 2018). This hypothesis is currently very general, as disaster severity can be measured in different ways among individuals and groups, including by duration of a single event, intensity or frequency/degree of exposure. Links between social capital and community resilience does not yet seem to be well understood across different types of disaster exposure contexts. It is difficult to assess what the role of social capital would have been if the damage was far worse on Gili Trawangan, as it was on neighboring Lombok, if social capital would have still been sufficient for a resilient response and recovery.

### Building social capital and community resilience

What can communities, governments, businesses or NGOs do to increase disaster preparedness? Gaps between knowing and doing are large, because social capital is an abstract concept built over time though specific community activities and actions, where context is important. As Lovell (2009) suggests “the major criticism facing social capital is a disconnect between social capital as a means of analysis and social capital as a policy goal”, (p. 781). What we know at least is that building social capital is a multidimensional process (Wilson 1997; Brewer 2003). On Gili Trawangan, two aspects were important. First, many activities and actions connected people before the earthquake, including the formation of community organizations such as GIDA and the Gili EcoTrust, establishing communication networks, joint financing, trust and reciprocity between many individuals. Second, activities and actions that create a sense of shared experiences and community identity were important. Examples before the earthquake include foreigners sharing experiences and knowledge about starting businesses abroad, dealing collectively with issues such as waste, government interactions or public transport difficulties from Bali, supply chains and hiring local staff. The island is also small and isolated, and it is easy to know who is living on the island (i.e., the in-group) and who isn’t (Partelow and Nelson 2018). The island’s nightlife scene also creates an open pro-social atmosphere. Of course, the earthquake itself was a shared experience for all. After the earthquake, explicit social media and community identity campaigns were developed under the slogan ‘Gili Strong’ (#GiliStrong), which more explicitly reflects the underlying sense of cognitive identity formed through the shared experience of the earthquake. Engaging in shared experiences, actions and activities seems to be a general feature that fosters social capital formation, mostly bonding and bridging ties. Either shared physical activities or cognitive shared experiences seem to be key, in ways that make sense in the community context e.g., (Taylor 2011; Gallagher et al. 2019; Li and Tan 2019; Shimpo et al. 2019). However, developing policies that do this in other places in ways that make sense locally, remains a substantial gap between what the literature says is beneficial and what can be done about it proactively and perhaps preventively for disaster resilience.

### Social capital, positionality and sustainability science

Conducting the interviews became a reflective process for many interviewees, who had otherwise not sat down and reflected on the events explicitly; either descriptively, or how they have been impacted psychologically or emotionally, or how they and the island had changed over the prior 3 months. Many interviewees stated that doing the interviews was helpful for them personally but also to reflect on what they had done together with others explicitly. Although I do not consider this study to be transdisciplinary by design, it is a useful example that warrants further reflection on how taking a problem and solution oriented sustainability science approach enacts a process of change through positionality, even if not intentionally. By asking questions and initiating reflection on social capital and collective action, I am also becoming embedded as an actor in that system influencing those processes. These issues warrant further examination in sustainability science, for example, recognizing that many forms of fundamental research can initiate real change processes in systems of study, even if they are not designed to do so. For example, in this study, does initiating explicit reflection on social capital and collective action in a community through the research process help enable its continued emergence?

As a researcher, I also bring my own biases to the interpretation and understanding of the events and data. My scientific training, scientific goals and cultural background being a few. Additionally, a challenging aspect of transcribing interviews into text, is the loss of voice tone, voice volume changes, speech pace changes, body language and setting. These play a large role in interpreting the meaning and perspective of the interviewee, which influence the analysis of what they say, but are difficult to convey in an article or to capture with current formal qualitative analytical methodologies, but nonetheless influence a researcher’s interpretation. In disaster research, it may be even more influential given the often traumatic nature of the events.

A final note on the importance of social capital studies in sustainability science. In science, we build social capital and communities to cooperate and enable our daily work and research, as much as the rest of the non-academic world does. However, building social capital between science and society is also an iterative and interdependent process and challenge as the field aims to advance transdisciplinary methods. A social capital perspective, as well as furthering social learning, communication and deliberation research, can be informative in applying what we know about other systems, the practices and principles, to ourselves and our research processes to achieve those goals.

## Electronic supplementary material

Below is the link to the electronic supplementary material.

Supplementary file1 (DOCX 27 kb)

## Footnotes

1 Measured by the USGS (2018) in Richter magnitude https://earthquake.usgs.gov/earthquakes/eventpage/us1000g3ub/executive.

2 https://www.kemsos.go.id/siaranpers/mensos-salurkan-santunan-556-korban-meninggal-gempa-ntb

3 A 7.5 magnitude earthquake and a devastating tsunami hit Palu, Sulawesi, Indonesia on September 28, 2018 causing over 4000 deaths, attracting far more international media attention than the earthquakes in Lombok.

4 It is a legal requirement that SCUBA businesses have Indonesian operators for their boats.

5 WhatsApp is a free digital text messaging platform, popular worldwide, particularly in Indonesia.
