# Supplementary material for: Social capital and community disaster resilience: post-earthquake tourism recovery on Gili Trawangan, Indonesia
Source: Sustain Sci. 2020 Sep 3;16(1):203–20. doi: 10.1007/s11625-020-00854-2 (PMC7471487; doi:10.1007/s11625-020-00854-2)
Supplement: Supplementary file 1 — Supplementary file1 (DOCX 27 kb) [file 11625_2020_854_MOESM1_ESM.docx]

**APPENDIX**

**Table S1.** Six dimensions of social capital from Jones and Woolcock (2007).

| **Components** | **Brief definitions** |
| --- | --- |
| (i) Groups and networks | “...enable people to access resources and collaborate to achieve shared goals…” |
| (ii) Trust and solidarity | “...the extent to which people feel they can rely on relatives, neighbors, colleagues, acquaintances, key service providers, and even strangers, either to assist them or (at least) do them no harm.” |
| (iii) Collective action and cooperation | “...whether and how people work with others in their community on joint projects and/or in response to a problem or crisis.” |
| (iv) Information and communication | “...the ways and means by which households receive and share information regarding such issues as the community at large, market conditions, and public services, as well as the extent of their access to communications infrastructure.” |
| (v) Social cohesion and inclusion | “...the tenacity of social bonds and their dual potential to include or exclude members of community.” |
| (vi) Empowerment and political action | “...the sense of satisfaction, personal efficacy, and capacity of network and group members to influence both local events and broader political outcomes.” |

**Table S2.** Description of the samples for both quantitative surveys, tourists and SCUBA managers/ owners.

| **Survey** | **Characteristics** | **Details** | **Amount** |
| --- | --- | --- | --- |
| Tourists | Overall sample | Tourist sample size in this study | 389 |
|  |  | Tourist population (per year) | ~1,000,000 |
|  | Age | Under 20 | 13 |
|  |  | 20-29 | 216 |
|  |  | 30-39 | 117 |
|  |  | 40-49 | 20 |
|  |  | 50-59 | 15 |
|  |  | 60-69 | 7 |
|  |  | 70 | 1 |
|  | Gender | Female | 170 |
|  |  | Male | 216 |
|  |  | N.A. | 3 |
|  | Nationality | Africa | 24 |
|  |  | Asia | 71 |
|  |  | Australia/ New Zealand | 37 |
|  |  | Europe | 185 |
|  |  | N.A. | 6 |
|  |  | North America | 47 |
|  |  | South America | 19 |
| SCUBA business managers/ owners | Sample | Sample size | 22 |
|  |  | Population size | 22 |
|  | Nationality of owners | Africa | 1 |
|  |  | Asia | 6 |
|  |  | Australia/ New Zealand | 1 |
|  |  | Europe | 15 |
|  |  | North America | 1 |
|  |  | South America | 2 |
|  | Year established  (# of SCUBA centers) | Before 2000 | 2 |
|  |  | 2000-2004 | 2 |
|  |  | 2005-2009 | 3 |
|  |  | 2010-2014 | 9 |
|  |  | After 2015 | 6 |
|  | Self-stated size of business compared to others on island | Large | 4 |
|  |  | Medium | 8 |
|  |  | Small | 10 |

**Table S3.** Consolidated criteria for reporting qualitative studies (COREQ): 32-item checklist.

| **No. Item** | **Guide questions/ description** | **Reported on Page #** | **Description from study** |
| --- | --- | --- | --- |
| **Domain 1: Research team and reﬂexivity** |  |  |  |
| *Personal Characteristics* |  |  |  |
| 1. Inter viewer/facilitator | Which author/s conducted the interview or focus group? | Results | [removed for blind peer-review] |
| 2. Credentials | What were the researcher’s credentials? E.g. PhD, MD | Methods | [removed for blind peer-review] |
| 3. Occupation | What was their occupation at the time of the study? | Methods | [removed for blind peer-review] |
| 4. Gender | Was the researcher male or female? | N/A | [removed for blind peer-review] |
| 5. Experience and training | What experience or training did the researcher have? | Methods | [removed for blind peer-review] |
| *Relationship with participants* |  |  |  |
| 6. Relationship established | Was a relationship established prior to study commencement? | N/A | This was the second study conducted on this case. Numerous interviewees had been interviewed in the previous/ first study in 2017. |
| 7. Participant knowledge of the interviewer | What did the participants know about the researcher? e.g. personal goals, reasons for doing the research | N/A | The interviewees knew the purpose of the research and gave informed verbal consent. Some interviewees knew the researcher from previous work, or had heard about him. |
| 8. Interviewer characteristics | What characteristics were reported about the inter viewer/facilitator? e.g. Bias, assumptions, reasons and interests in the research topic | Methods | The interested in the research topic was conveyed to the interviewees. |
| **Domain 2: study design** |  |  |  |
| *Theoretical framework* |  |  |  |
| 9. Methodological orientation and Theory | What methodological orientation was stated to underpin the study? e.g. grounded theory, discourse analysis, ethnography, phenomenology, content analysis | Methods | Qualitative content analysis  Ethnography |
| *Participant selection* |  |  |  |
| 10. Sampling | How were participants selected? e.g. purposive, convenience, consecutive, snowball | Methods | Purposive/ key-informant  Snowball |
| 11. Method of approach | How were participants approached? e.g. face-to-face, telephone, mail, email | Methods | Face-to-face  Email  Whatsapp (texting) |
| 12. Sample size | How many participants were in the study? | Results | 44 |
| 13. Non-participation | How many people refused to participate or dropped out? Reasons? | Methods | 0 |
| *Setting* |  |  |  |
| 14. Setting of data collection | Where was the data collected? e.g. home, clinic, workplace | Methods | Gili Trawangan, Indonesia  Public places (cafes, restaurants, SCUBA centers, beach) |
| 15. Presence of non-participants | Was anyone else present besides the participants and researchers? | Results | ~25% of the interviews were accompanied by a master’s student conducting a separate research project in the area. |
| 16. Description of sample | What are the important characteristics of the sample? e.g. demographic data, date | Results | This data is provided in the appendix. |
| *Data collection* |  |  |  |
| 17. Interview guide | Were questions, prompts, guides provided by the authors? Was it pilot tested? | Methods | Yes, general guiding questions were developed in advance of the interviews. |
| 18. Repeat interviews | Were repeat inter views carried out? If yes, how many? | N/A | Yes, four. With these individuals, either the initial interview was not enough time to ask/ answer all questions, or the I approached them with follow up questions. |
| 19. Audio/visual recording | Did the research use audio or visual recording to collect the data? | Methods | No. |
| 20. Field notes | Were ﬁeld notes made during and/or after the inter view or focus group? | Methods | Detailed field notes were made during and after each interview. |
| 21. Duration | What was the duration of the inter views or focus group? | Methods | Interviews ranged from 20min to 2 hours. |
| 22. Data saturation | Was data saturation discussed? | Methods | Yes. |
| 23. Transcripts returned | Were transcripts returned to participants for comment and/or correction? | N/A | No. |
| **Domain 3: analysis and ﬁndings** |  |  |  |
| *Data analysis* |  |  |  |
| 24. Number of data coders | How many data coders coded the data? | Methods | One |
| 25. Description of the coding tree | Did authors provide a description of the coding tree? | N/A | Social capital framework. |
| 26. Derivation of themes | Were themes identiﬁed in advance or derived from the data? | Methods | Both. A general framework was used to guide questions for data collection (Jones and Woolcock, 2007), and a second framework ‘Three types of social capital’ to guide data organization and analysis (Szreter and Woolcock, 2004; Kawachi et al., 2004; Newman and Dale, 2007; Aldrich and Meyer, 2015). Within the analysis framework, inductive themes were derived. |
| 27. Software | What software, if applicable, was used to manage the data? | Methods | MaxQDA |
| 28. Participant checking | Did participants provide feedback on the ﬁndings? |  | No |
| *Reporting* |  |  |  |
| 29. Quotations presented | Were participant quotations presented to illustrate the themes/ﬁndings? Was each quotation identiﬁed? e.g. participant number | Results | Quotations in the text are used to represent the main themes of the analysis. Each quote is labelled by the anonymized stakeholder group code for reference. |
| 30. Data and ﬁndings consistent | Was there consistency between the data presented and the ﬁndings? |  | Data supported the hypotheses in this study. |
| 31. Clarity of major themes | Were major themes clearly presented in the ﬁndings? | Results | All major themes from the conceptual framework used, were identified empirically. |
| 32. Clarity of minor themes | Is there a description of diverse cases or discussion of minor themes? | Discussion | The discussion section links to other literature in the field and discusses the findings of the study in relation. |

**Table S4.** General guiding questions used for interviews. Not all questions were asked for every interviewee. This list was compiled after all interviews, from guiding questions developed in a field notebook before each interview. These questions aim to give an indication and do not represent a comprehensive list of all questions asked given the semi-structured nature of the interviews.

| **Aggregated general questions**  What is your full name and affiliation?  What previous organizations / businesses have you worked for or been involved with?  How long have been living/ working on the island?  Where were you during the earthquake?  What did you do in the minutes, hours, days after the earthquake?  What did others you know do?  Who did you interact with following the earthquake?  Can you describe the damage/ impact of the earthquake on the island?  How were you affected by the earthquakes?  Do you feel you can rely on the Gili Trawangan community?  What are the main social challenges on the island?  How has the recovery process been for the island after the earthquake?  What has changed on the island since the earthquakes?  Do you think the island will recover from the earthquake?  **Aggregated specific questions (depending on individual)**  What happened in your island group during the closure period?  What happened in your Bali group during the closure period?  How was your business/ organization involved in the relief aid for Lombok?  How important are the Gili islands for Lombok?  What is your assessment of the relationship between local Indonesian staff and foreigners?  How you think the earthquake will influence tourism?  What is the best way for the island to recover (tourism economy)?  How would describe your relationship with employees?  How would do describe your relationship with local Indonesians?  Do you think there was a difference in how local Indonesians vs foreigners were affected by the earthquake?  Do you think there was a difference in how local Indonesians vs foreigners interacted after the earthquake than before?  How was your business affected?  Has your business recovered?  How has your life changed after the earthquake?  Did the island receive any external aid?  What is the relationship between local businesses and the local and national government? |
| --- |
